# Outcome of diabetic ketoacidosis among paediatric patients managed with modified DKA protocol at Tikur Anbessa specialized hospital and Yekatit 12 hospital, Addis Ababa, Ethiopia

**DOI:** 10.1002/edm2.363

**Published:** 2022-08-09

**Authors:** Tigist Bacha, Yemisrach Shiferaw, Ermias Abebaw

**Affiliations:** ^1^ Department of Pediatrics and Child Health, School of Medicine, College of Health Sciences St Paul Millennium Medical College Addis Ababa Ethiopia; ^2^ Department of Pediatrics and Child Health, School of Medicine, College of Health Sciences Addis Ababa University Addis Ababa Ethiopia; ^3^ Department of Pediatrics and Child Health, School of Medicine Debre Markos University Debre Markos Ethiopia

**Keywords:** diabetic, ketoacidosis, modified, outcome, protocol

## Abstract

**Introduction:**

Diabetic ketoacidosis (DKA) is a serious acute complication of diabetes mellitus that carries a significant risk of mortality with delayed treatment in low‐resource countries. This study aimed to determine the outcome of paediatric DKA patients' managed with a modified DKA treatment protocol using intermittent bolus subcutaneous insulin administration.

**Methods:**

A cross‐sectional study design with retrospective data collection was conducted among children younger than 14 years of age admitted from January 2013 to February 2017. A modified protocol was prepared based on a reference from the international society for paediatric and adolescent diabetes and other international guidelines. Data were analysed using Statistical package for social science (SPSS) version 22.0. Descriptive statistics were performed. Binary logistic regression was used to identify associations, and significant variables were further considered for multivariate logistic regression to determine the outcome of DKA patients.

**Result:**

Among the 190 patients, 55.5% (*n* = 105) were newly diagnosed. The overall average time required for resolution of DKA was 48 ± 27.8 h. Mental status on presentation (*p* = .001), shock on presentation (*p* < .01) and severity of DKA (*p* < .001) were found to have a significant association with the mean time for clearance of DKA. Hypoglycaemia was the most common treatment‐related complication, which occurred in 23.7% of patients (*n* = 45) followed by hypokalaemia in 4.3% of patients (*n* = 8), and no patient developed cerebral oedema and death.

**Conclusion:**

The time required for clearance of DKA was prolonged, and hypoglyceamia was a common complication for children younger than 5 years of age. The modified protocol of DKA is reasonable management for low‐resource settings with further modification.

## Graphical abstract

Time required for clearance of DKA was prolonged and hypoglycemia was a common complication for under five children. The modified protocol of DKA is reasonable management for low resource setting with further modification for under five children.

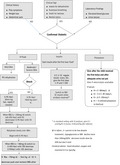

## 1 BACKGROUND

Diabetic ketoacidosis (DKA) is a life‐threatening condition associated with type 1 diabetes that necessitates repeated admissions in children.^1^ There is wide geographic variation in the frequency of DKA at the onset of diabetes; rates inversely correlate with the incidence of type 1 diabetes. It is reported in 1%–10% of known type 1 diabetic patient in industrialized countries, while it occurs in 35%–40% of such patients in Africa. DKA at the time of the first diagnosis of diabetes mellitus is reported in 15%–70% in Western Europe and North America, but is seen in 95% of diabetic children in African countries, and frequency reaches up to 80% in a study done in Ethiopia.^2^, ^3^, ^4^, ^5^, ^6^

A high index of clinical suspicion is necessary to diagnose DKA, especially in a younger child because it is difficult to obtain the classical polysymptoms such as polydipsia and polyuria.^7^, ^8^, ^9^

The criteria to diagnose DKA in children include: blood glucose >200 mg/dl, venous pH <7.3, or bicarbonate <15 mmol/L and ketonemia with ketonuria. The severity is also assessed and categorized by the degree of acidosis as mild, moderate or severe.^8^, ^10^, ^11^

Short of available biochemical tests, the diagnosis of DKA mainly relies on the presence of hyperglycaemia which is blood glucose >200, glycosuria and ketonuria in resource‐limited set‐ups. The severity is also assessed based on the clinical condition of the child as mild if the patient is oriented, alert but fatigued with signs of no to some dehydration; moderate if Kussmaul respiration is present, has signs of dehydration, and is sleepy; and sever if Kussmauls or depressed respiration is present, has signs of severe dehydration or shock and has depressed sensorium to coma.^12^, ^13^

The critical factors in the treatment of DKA include correcting dehydration, correcting acidosis and reversing ketosis, restoring blood glucose to near normal, monitoring for complications and treating any precipitating event.^8^, ^14^

Management of dehydration is always the priority in DKA patients. Volume expansion with 10–20 ml/kg of 0.9% saline over 1 h is required, daily maintenance fluid requirement and the estimated fluid deficit which is 8.5% is calculated to be given over 48 h. In rare instances, patients with shock need 20 ml/kg boluses of isotonic saline. Deficit replacement should be with normal saline with added 40 meq/L of potassium chloride, and further adjustment of fluid is based on serum random blood sugar (RBS). Normal saline will be given until RBS is 250–300, and fluid will be changed to dextrose in normal saline (DNS) or ½ normal saline(NS) in 5% dextrose in water (DW) when the RBS drops to 140–250, 1/2 NS in 10% dextrose will be administered when RBS < 140 and 10% IV push if RBS < 70.^12^, ^14^, ^15^, ^16^, ^17^

If a patient has repeated hypoglycaemia, the rate of glycemic drop is more than 100 mg/dl/hour or hypokalaemia unresponsive to other management, the dose of insulin is decreased by half but never discontinued.^12^, ^13^ Patients with mild DKA usually do not have impaired peripheral circulation and, therefore, do not require a fluid bolus.^12^, ^14^, ^15^, ^16^, ^17^

Insulin administration is started 1–2 h after starting fluid replacement therapy. Continuous intravenous regular insulin (CIRI) at a rate of 0.1 IU/Kg/h until resolution of DKA is commonly used. On the other hand, a lower intravenous regular insulin dose of 0.05 IU/kg/hour can be safe and effective.^8^ In circumstances where CIRI administration is not possible, hourly or 2 hourly subcutaneous (SC) or intramuscular (IM) administration of insulin may be as effective as intravenous (IV) regular insulin infusion but subcutaneous (SC) should not be used in patients whose peripheral circulation is impaired.^13^, ^18^, ^19^, ^20^, ^21^, ^22^

The international society for paediatrics and adolescent medicine has set guidelines for the management of DKA which is currently being practised in most countries. In our set‐up, there was no standard of practice in the management of DKA until the modified DKA management protocol was adopted in 2014 and put into practice. This protocol was developed from the international guideline, standard textbooks and ISPAD; incorporated aiming to have a standard of care and to improve the outcome of paediatric DKA patients.

According to this modified DKA protocol, fluid is given for severe and moderate DKA as per the above protocol except for the management of mild DKA which could be managed with oral rehydration solution, and deficit calculated as some dehydration and 75 ml/kg of fluid is given and patients are encouraged to take PO feeding.^12^, ^13^

**FIGURE 1:**
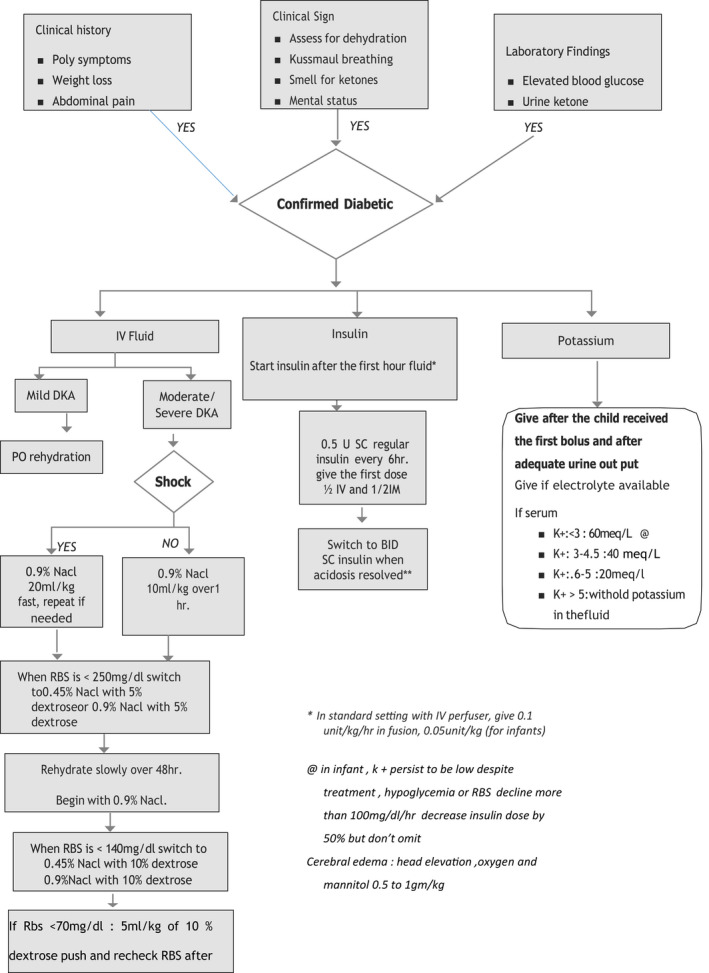
Management algorithm for diabetic ketoacidosis in children in Ethiopia [Correction added on 12 September 2022 after first online publication: In figure 1, the word 'recheck' and '10%' were updated in the left last box.]

As there is a lack of readily available perfuser, no strict follow‐up of patients with frequent monitoring of RBS and no readily available intensive care in Ethiopia, intermittent SC regular insulin is used in place of Continuous intravenous regular insulin (CIRI); with a loading dose of 0.5 units/kg of regular insulin given in divided doses as half IV and half IM and then 0.5 IU/kg SC every 6 h except the first dose which is administered ½ IM and ½ IV since dehydration in the first hour limits absorption of subcutaneous insulin, and it is then continued until resolution of DKA; that is, two consecutive urine ketones negative. Follow‐up of a patient is with a DKA flow sheet (Figure 1).

Even though we currently have a standard of care, the protocol is not supported by previous proven evidence, and also no study was done so far to see the effectiveness as well as the treatment outcome of patients managed with this treatment protocol. Therefore, this study aimed to determine the outcomes of patients managed with the modified DKA treatment protocol used at TASH and Yekatit 12 Hospital.

## 2 METHODS

### 2.1 Study design, period and population

An institution‐based cross‐sectional design with a retrospective chart review was conducted in two hospitals Tikur Anbessa Specialized Hospital (TASH) and Yekatit 12, both located in Addis Ababa, Ethiopia. TASH has about 600 beds and offers diagnosis and treatment for approximately 400,000 patients a year. The paediatric emergency and critical care unit gives 24‐hour service; has 42 beds; and is staffed with two emergency and critical care trained consultants, residents, interns and nurses while the paediatric ICU has four beds with only two perfusers. Yekatit 12 Hospital gives both teaching and clinical service. The paediatric emergency unit functions 24 h and is staffed with general paediatricians, residents, interns and nurses. In the time of study, there was no paediatric intensive care unit, no perfusers, blood gas analysis and serum ketone test services. All paediatric DKA patients are initially evaluated at the paediatric emergency unit and then admitted and managed in the emergency ward, inpatient wards or rarely in the paediatric ICU. The study population included patients <14 years who were admitted to TASH and Yekatit 12 hospital from January 2013 to February 2017 with the diagnosis of DKA.

### 2.2 Inclusion criteria

All paediatric patients less than 14 years, admitted to TASH and Yekatit 12 Hospital with DKA from January 2013 to February 2017.

### 2.3 Exclusion criteria

All patients without full records, all patients admitted to the ICU and managed via perfuser.

### 2.4 Sample size determination and sampling technique

A total of 190 participants who fulfilled the eligibility criteria during the study period were taken as our study population.

### 2.5 Data collection procedure

The chart number of patients admitted at the emergency ward of Yekatit 12 hospital and TASH from January 2013 to February 2017 was retrieved from the emergency ward log book of both hospitals, and selection was made according to the sampling procedure. Data were entered into a prepared checklist and a general practitioner filled in the required data.

### 2.6 Data processing and analysis

Data were entered into the computer using epi info and exported into SPSS version 22 for analysis. Descriptive statistics was performed using frequency distribution, mean, median and standard deviation. To see the association between categorical variables, binary logistic regression was used, and the odds ratio was calculated to determine the risk of developing hypoglycaemia. ANOVA 1 test was used to determine the mean difference in time for resolution of DKA among selected independent variables. *p*‐value < .05 was considered statistically significant. No comparison was made between the two hospitals because it is assumed that similar care is provided.

### 2.7 Operational definition

*Diabetic history*: Whether the patient is newly diagnosed or a known patient with type 1 DM.

*Length of hospital stay*: Time between admission and discharge date.

*Mild DKA*: Patient who fulfils criteria for DKA and has fatigue, has no sign or has sign of some dehydration and no Kussmaul's breathing.

*Moderate DKA*: Patient who fulfils criteria for DKA and has fatigue, sleepiness, sign or symptom of dehydration and Kussmaul's breathing.

*Severe DKA*: Patient who fulfils criteria for DKA with a sign or symptom of dehydration or shock, Kussmaul's breathing and altered sensorium to coma.

*Hypoglycaemia*: RBS level less than 70.

*Outcome*: Includes primary and secondary outcomes.

- *Primary outcome*: Time required for clearance of DKA (prolonged vs not prolonged).
- *Secondary outcome*: Presence versus absence of complication including hypoglycaemia, hypokalaemia, cerebral oedema and death due to DKA or management complication which occurs after admission and before the discharge of the patient.

*The time required for resolution of DKA*: Is calculated from the time at which management of DKA is started to the time management of DKA is discontinued.

*Time for resolution of DKA is prolonged*: If >12 h for a patient with mild DKA and >36 h if moderate or severe DKA.

## 3 RESULT

### 3.1 Demographic and baseline clinical characteristics of patients

A total of 270 patients were admitted with the diagnosis of DKA in both hospitals over the 4‐year study period. From this, 194 were randomly selected and included in the study because four did not meet inclusion criteria. Among the 190 participants, 55.5% (*n* = 105) were newly diagnosed and 44.5% (*n* = 85) where known DM patients. The median age of presentation was 8 years; 42.6% (*n* = 81) of the patients were males Family history of DM was present in 14.2% (*n* = 27). Polysymptom was present in 73.2% (*n* = 139) and 8.4% (*n* = 16) presented with shock (Table 1).

**TABLE 1:** Demographic feature and baseline clinical characteristics of paediatric patients with DKA admitted to TASH and Yekatit 12 Hospital from January 2013 to February 2017, Addis Ababa, Ethiopia (*n* = 190)

Variable	Category	Number (*n*)	Percentage
Sex	Male	81	42.6
	Female	109	57.4
Age	<1 year	5	2.6
	1–5 year	60	31.6
	>5 year	125	65.8
Address	Addis Ababa	161	84.7
	Out of Addis Ababa	29	15.3
Diabetic History of the patient	New	105	55.8
	Known	84	44.2
Clinical Feature	Poly symptoms	139	73.2
	Vomiting	93	48.9
	Weight loss	31	16.3
	Fatigue	51	26.8
	Abdominal pain	49	25.8
	Fever	20	10.5
	Difficulty breathing	36	18.9
	Change in mentation	24	12.6
Family history of DM	Yes	27	14.2
	No	130	68.4
	Unknown	33	17.4
Shock	Yes	16	8.4
	No	174	91.6

### 3.2 The severity of DKA and precipitating factor

**FIGURE 2:**
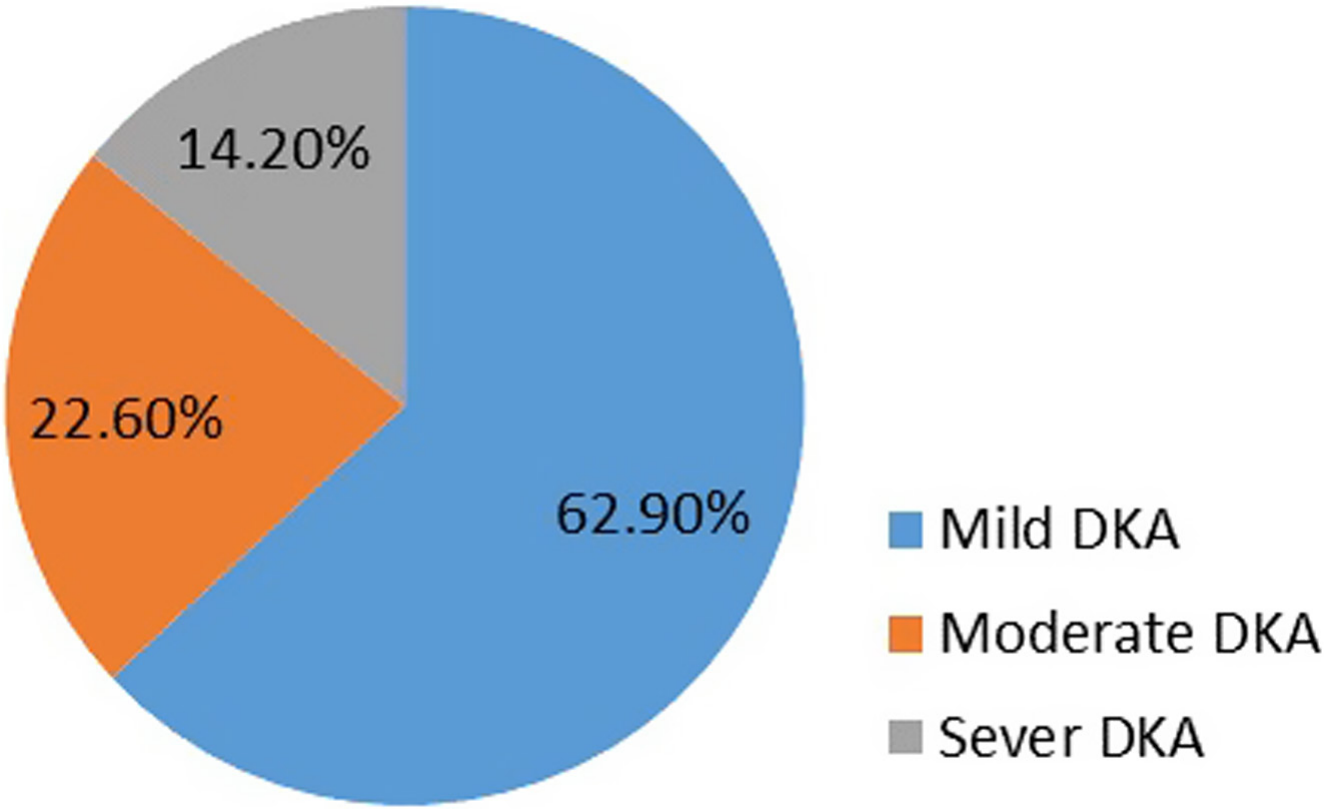
Severity of DKA among paediatric patients admitted to TASH and Yekatit 12 Hospital, Addis Ababa, Ethiopia

Among the total patients, 62.9% (*n* = 119) had mild DKA, 22.6% (*n* = 43) moderate and 14.7% (*n* = 28) severe DKA (Figure 2). Infection was the most common precipitating factor in 24.3% (n = 46); the associated infections being pneumonia (*n* = 18), urinary tract infection (*n* = 14), acute gastroenteritis (*n* = 7), upper respiratory tract infection (*n* = 6) and meningitis (*n* = 1). Drug discontinuation was the second common precipitating factor 16.3% (*n* = 31), and no precipitating factor was identified in 52% (*n* = 99) (Table 2).

**TABLE 2:** Outcome of DKA paediatric patients managed with the modified DKA protocol admitted to TASH and Yekatit 12 Hospital from January 2013 to February 2017, Addis Ababa, Ethiopia (*n* = 190)

Variable	Mean Time(in hour)	Std. Deviation
Time required for clearance of DKA		
Mild DKA	42.71	25.68
Moderate DKA	49.77	26.59
Severe DKA	68.07	30.77

Variable	The median length of stay(in day)	Min and max.
Length of stay in the hospital		
Overall length of stay	7	2–20

Variable	Number	Percent
Complication of DKA management		
Hypoglycaemia	45	23.7
Hypokalemia	8	4.3
Cerebral oedema	‐	‐
Death	‐	‐

### 3.3 Outcome

All of the patients were managed according to the modified DKA protocol, with a requirement of change of fluid in 34.2% (*n* = 65) and a decrement of insulin by half due to recurrent hypoglycaemia in 3.2% (*n* = 6). The overall average median time required for resolution of DKA was 44.5 h with Inter Quartile Range (IQR) of 24.75–65.25 and specifically for patients with mild DKA 42.7 ± 25.68 h, for moderate DKA 49.7 ± 26.5 h and severe DKA 68 ± 30.7 h.

Hypoglycaemia was the most common treatment‐related complication, which occurred in 23.7% of patients (*n* = 45) and hypokalaemia occurred in 4.3% of patients (*n* = 8). No patient developed cerebral oedema and no death occurred in patients managed with this protocol. The median length of hospital stay of patients was 7 days with a range of 2–20 days (Table 3).

**TABLE 3:** Mean time for resolution of DKA for different independent variables among paediatric patients admitted to TASH and Yekatit 12 Hospital from January 2013 to February 2017, Addis Ababa, Ethiopia (*n* = 190)

Variable		Time for clearance of DKA			
		Mean(hour)	Std. Deviation	95% CI	*p* Value
Age	<1	62.20	23.690	32.79–91.61	.270
	1–5	50.90	27.342	43.84–57.96	
	>5	45.95	28.123	40.97–50.93	
Diabetic History	Newly diagnosed	51.20	28.527	45.6–56.728	.073
	Known	43.87	26.733	38.07–49.67	
Mental status on presentation	Alert	43.82	25.224	39.62–48.02	.001
	Lethargic	55.97	30.287	45.57–66.38	
	Comatose	69.36	34.112	49.66–89.05	
Shock on presentation	No	25.676	1.946	6–140	<.001
	Yes	34.677	8.669	27–138	
Severity of DKA	Mild	42.71	25.681	38.05–47.38	<.001
	Moderate	49.77	26.588	41.58–57.95	
	Severe	67.36	30.427	55.56–79.16	

### 3.4 Comparison of mean time for resolution of DKA and different categorical independent variable

ANOVA one test was used to see an association between mean time for resolution of DKA and different independent variables and to compare mean difference. Mental status (*p* = .001), shock on presentation (*p* = .001) and severity of DKA (*p* = .001) were found to have a significant association with the mean time for clearance of DKA. The median time for resolution of DKA in a comatose patient was 25.53 h more than that of an alert patient (*p* = .003); that of a lethargic patient was 12.14 h more than the alert patient (*p* = .043), and the mean time for DKA resolution in a patient with severe DKA was 24.64 h more than in mild DKA (*p* = .001), and in severe DKA was 17.59 h more than in moderate DKA (*p* = .021); the meantime for resolution of DKA in a patient with shock was 31.32 h more than that of with no shock (*p* = .001) (Table 4).

**TABLE 4:** Factors associated with hypoglycaemia among paediatric patients admitted to TASH and Yekatit 12 Hospital from January 2013 to February 2017, Addis Ababa, Ethiopia (*n* = 190)

Variable		Hypoglycaemia		*p*‐Value	AOR (95% CI)
		No	Yes		
Age	<1 year	2	3	.000	9.529 (1.482–16.266)
	1–5 year	35	25		4.538 (2.199–9.365)
	>5 year	108	17		Ref.
Kussmaul's breathing	No	104	19	.000	Ref.
	Yes	41	26		3.471(1.718–14.135)
Shock	No	138	36	.003	Ref.
	Yes	7	9		4.929 (1.718–14.135)
DKA severity	Mild	101	18	.000	Ref.
	Moderate	32	11		1.929 (0.825–4.508)
	Severe	12	16		7.481 (3.039–18.418)

### 3.5 Association between hypoglycaemia and different independent variables

Binary logistic regression was used to identify the association between hypoglycaemia and the categorical independent variables, and the odds ratio was used to determine the risk of developing hypoglycaemia in each category for those with significant associations. Age less than 1 year (*p* = .001), shock on presentation (*p* = .003), Kussmaul's breathing (*p* = .001) and DKA severity (*p* = .001) were found to have a statistically significant association with the development of hypoglycaemia.

Age less than 1 year and age 1–5 years have 9.5 times and 4.5 times higher risk of developing hypoglycaemia as compared with age group >5 years (AOR 9.53 [1.48–16.27] and 4.54 [2.11–9.36], respectively). Patients with Kussmaul's breathing on presentation have a 3.5 times higher risk of developing hypoglycaemia as compared with those with no Kussmaul's breathing (AOR = 3.47[1.72–14.14]). Patients with shock on presentation have a 4.9 times higher risk of hypoglycaemia as compared with those with no shock (AOR = 4.93 [1.72–14.14]). Patients with severe DKA have a 7.5 times higher risk of developing hypoglycaemia during treatment as compared with those with mild and moderate DKA (AOR = 7.48 [3.04–18.42]) (Table 4).

## 4 DISCUSSION

This study was done to see the outcome of paediatric DKA patients managed with the modified protocol at TASH and Yekatit 12 hospital. Based on our study, the time required for clearance of DKA was prolonged, and hypoglycaemia was a common complication for children younger than 5 years of age.

The mean age of presentation and sex distribution with slight female predominance in our study was similar to findings of research done in TASH and Costa Rica.^2^, ^4^, ^23^ The median time of presentation was shorter than the findings of research done in TASH and Costa Rica.^2^, ^23^ This could be due to increased awareness of the attendants because the percentage of known DM patients included in this study is higher in this study.

Polysymptom was the predominant presentation which is consistent with research done in TASH and Costa Rica.^2^, ^23^ This is in contrast to the study done in Saudi Arabia and Pakistan.^3^, ^27^ The difference might be attributable to the proportion of age above 5 years and newly diagnosed patients being high in our study.

Family history of DM was smaller as compared with findings of a study done in Nigeria and Saudi Arabia.^26^, ^27^ This is probably because the patient's attendant might not remember and provide the right information because of recall bias.

Infection was a common precipitating factor followed by drug discontinuation; this is consistent with the finding of Costa Rica and Saudi Arabia.^23^, ^27^

In this study, the overall average time required for resolution of DKA was 48 ± 27.8 h and specifically for patients with mild DKA 42.7 ± 25.7 h, moderate DKA 49.7 ± 26.5 h and for severe DKA 68.1 ± 30.8 h which was longer than the findings of research done in Costa Rica which was 7.8 days, 40.6 ± 12.8 in PICU for patient with established diabetes and 54.1 ± 15.55 for patient with newly diagnosed diabetes with DKA in Pakistan and 3.26 days in the PICU in Indonesia.^23^, ^29^, ^31^ This could be because of the difference in management protocol and clearance of urine ketone was used to diagnose the resolution of DKA while others used serum ketone biochemical markers.

Hypokalaemia and cerebral oedema were lower in this study in contrast to studies done in Iran, Saudi Arabia, India and Pakistan.^25^, ^27^, ^28^ Hypokalaemia occurred in 4.3% of our patients and no patient developed cerebral oedema. No death occurred during the study period. Hypoglycaemia was the most common treatment‐related complication and occurred in 23.7% of patients which is a higher number compared with research done in other set‐ups.^24^, ^25^, ^26^, ^27^, ^28^, ^29^ This could probably be due to a lack of strict follow‐up to the rate of drop in RBS, and it could also be because of the bolus insulin administered during the initial management.

Age of the patient, shock on presentation, Kussmaul's breathing and DKA severity were found to have a statistically significant association with the development of hypoglycaemia. Those aged less than 5 years have a higher risk of developing hypoglycaemia as compared with age group >5 years. Patients with Kussmaul's breathing on presentation have a higher risk of developing hypoglycaemia as compared with those with no Kussmaul's breathing.

The median length of hospital stay of patients was 7 days which was lesser than studies done in TASH, Costa Rica and Pakistan.^2^, ^8^, ^28^ This is probably because the number of known diabetic patients included in our study is higher as compared with previously cited studies, and hence, patients with known diabetes presented with DKA do not need to stay in the hospital for diabetic education.

Mental status on presentation, shock and severity of DKA were associated with the mean time for clearance of DKA. This finding is consistent with a study in India and Kenya.^25^, ^30^ This is explained by coma on presentation which might be associated with cerebral oedema and severe DKA. The presence of those factors may require longer hospital stays and treatment that leads to prolonged DKA clearance.

## 5 CONCLUSION

The time required for the resolution of DKA is prolonged in paediatric DKA patients managed with modified protocol in this study. Mental status on presentation, shock on presentation and severity of DKA were found to have a significant association with the mean time for resolution of DKA. Hypoglycaemia was a common management‐related complication in patients managed with modified DKA protocol. Patients' age, Kussmaul's breathing, shock and severe DKA had a higher risk of developing hypoglycaemia. Hypokalaemia and cerebral oedema were not common treatment‐related complications and no death occurred. Modified DKA score works best and needs modification for children younger than 5 years of age to decrease the risk of developing hypoglycaemia. Frequent monitoring of RBS in the groups identified to have a higher risk of hypoglycaemia is recommended.

The retrospective nature of this study made the study difficult to determine the average hourly drop in serum glucose level and frequency of adjustment of fluid. Secondly, short of serum ketone and blood gas analysis, it is difficult to exactly determine the clearance of ketone and resolution of DKA which is another setback of this study. Moreover, the exact time of complication occurred was challenging to trace from the charts.

## AUTHOR CONTRIBUTIONS

**Tigist Bacha:** Conceptualization (lead); data curation (supporting); formal analysis (lead); methodology (lead); software (lead); supervision (lead); writing – review and editing (lead). **Ermias Abebaw:** Formal analysis (supporting); software (supporting); writing – review and editing (lead). **Yemisrach Shiferaw:** Conceptualization (lead); data curation (lead); formal analysis (lead); investigation (lead); methodology (lead); software (lead); supervision (lead); writing – original draft (lead).

## CONFLICT OF INTEREST

The authors have declared that they have no competing interest.

## Data Availability

The data sets used and/or analysed during the current study are available from the corresponding author on reasonable request.
